# Tartary Buckwheat R2R3-MYB Gene *FtMYB3* Negatively Regulates Anthocyanin and Proanthocyanin Biosynthesis

**DOI:** 10.3390/ijms23052775

**Published:** 2022-03-03

**Authors:** Lei Wang, Renyu Deng, Yuechen Bai, Huala Wu, Chenglei Li, Qi Wu, Haixia Zhao

**Affiliations:** College of Life Science, Sichuan Agricultural University, No. 46, Xinkang Road, Ya’an 625014, China; leiwang@sicau.edu.cn (L.W.); dengrenyu@caas.cn (R.D.); ybai@ice.mpg.de (Y.B.); hualawu@sicau.edu.cn (H.W.); 13981@sicau.edu.cn (C.L.); wuqi@sicau.edu.cn (Q.W.)

**Keywords:** anthocyanin, FtMYB3, proanthocyanidin, Tartary buckwheat, transcription factor

## Abstract

Anthocyanins and proanthocyanidins (PAs) are vital secondary metabolites in Tartary buckwheat because of their antioxidant capacities and radical scavenging functions. It has been demonstrated that R2R3-MYB transcription factors (TFs) are essential regulators of anthocyanin and PA biosynthesis in many plants. However, their regulatory mechanisms in Tartary buckwheat remain to be clarified. Here, we confirmed the role of FtMYB3 in anthocyanin and PA biosynthesis. FtMYB3, which belongs to the subgroup 4 R2R3 family was predominantly expressed in roots. The transcriptional expression of FtMYB3 increased significantly under hormone treatment with SA and MeJA and abiotic stresses including drought, salt, and cold at the seedling stage. Functional analyses showed that FtMYB3 negatively regulated anthocyanin and PA biosynthesis, primarily via downregulating the expression of the *DFR*, *ANS*, *BAN*, and *TT13* in transgenic *Arabidopsis thaliana*, which may depend on the interaction between FtMYB3 and FtbHLH/FtWD40. Altogether, this study reveals that FtMYB3 is a negative regulatory transcription factor for anthocyanin and PA biosynthesis in Tartary buckwheat.

## 1. Introduction

Anthocyanins and proanthocyanidins (PAs) are derived from phenylalanine and mainly distributed in the fruit, bark, leaves and seeds of plants [1]. They have multiple functions in plants, including attraction of pollinators, protection against UV light damage and oxidative stress, regulation of auxin transport and allelopathy [2,3]. Moreover, PAs and anthocyanins show different health effects in vitro, such as anticancer and anti-inflammatory activities, as well as antioxidation [4,5]. Therefore, further exploring their synthesis and regulatory mechanisms is essential.

To date, increasing evidence suggests that the anthocyanin and PA biosynthetic pathway is well-conserved in monocots and dicots, and most steps are the same [6,7]. Firstly, leucoanthocyanidins are converted into unstable anthocyanidins by anthocyanidin synthase (ANS). Then, anthocyanidins are either immediately glycosylated to anthocyanins by anthocyanidin 3-O-glycosyltransferases (UGTs) or reduced to flavan-3-ols by anthocyanidin reductase (ANR) for PA biosynthesis [8]. Previous studies have elucidated that the above process is mainly regulated by TFs such as MYB, bHLH, WD40, and WRKY families [9,10,11]. Among these TFs, R2R3 MYB plays an indispensable role in regulating anthocyanin and PA biosynthesis, which have been studied extensively in different species. For example, several activators, including PcMYB114, McMYB12, MdMYB9/11, co-regulate PA and anthocyanin biosynthesis [12,13,14]. In contrast, NtMYB3 and MdMYB16 inhibit anthocyanin biosynthesis via the typical conserved motifs C2 at their C-terminus [15,16]. However, an R2R3-MYB TF, VviMYB86 positively regulated PA biosynthesis by upregulating the *LAR* expression but repressed the anthocyanin biosynthesis with downregulation of the transcript levels of *VviANS* and *VviUFGT* [17]. These studies indicate that the molecular mechanism underlying anthocyanin and PA accumulation is complicated. Notably, the ternary complex of MYB proteins is also involved in the synthesis of anthocyanins and PAs in different plants [18,19]. Recently, two novel ternary complexes, the JAZ1-TRB1-MYB9 and MdHY5-MdWRKY41-MdMYB were found to dynamically modulate anthocyanin and PA accumulation in apple [20,21]. In addition, anthocyanin and PA biosynthesis are also influenced by environmental signals such as phytohormone signals, UV radiation, cold, drought and light [22,23]. Collectively, this strongly suggests that MYB TFs play a central role in anthocyanin and PA synthesis.

Tartary buckwheat (*Fagopyrum tataricum*), which belongs to the genus *Fagopyrum* in the Polygonaceae family, is widely recognized as a pseudo-cereal that possesses unparalleled nutritive value [24,25]. As a plant originating in high-altitude areas, abiotic stresses such as ultraviolet and drought have a significant effect on the growth of Tartary buckwheat. Studies have reported that the accumulation of anthocyanins and PAs has benefits for plant survival under these stresses [26,27,28]. Therefore, understanding the corresponding mechanisms of their biosynthesis and regulation in Tartary buckwheat has gained considerable attention. Recently, several positive and negative regulators were identified in Tartary buckwheat. The first functional characterization of R2R3-type MYB TFs from Tartary buckwheat, found that FtMYB1 and FtMYB2 control the PA accumulation [29]. Ectopic expression of *FtMYB15* in *Arabidopsis* promotes the accumulation of anthocyanins and PA in leaves and seed coats [30]. The latest studies showed that FtMYB8 and FtMYB18 from the SG4-like MYB subfamily act as a negative regulator of anthocyanin/PA biosynthesis [31,32]. Additionally, our previous study showed that FtMYB3 interacted with the jasmonate-ZIM domain protein FtJAZ2 to influence anthocyanin biosynthesis in Tartary buckwheat [33]. Furthermore, FtMYB3 is likely to be involved in anthocyanin biosynthesis in the hairy roots of Tartary buckwheat [34]. However, the molecular mechanism of FtMYB3 mediating in which signaling pathways of the anthocyanin biosynthesis remains unclear. In this study, we validated that FtMYB3 acts as a negative regulator of anthocyanin and PA accumulation. This study sheds light on the regulatory mechanism of R2R3-MYB repressors on anthocyanin and PA synthesis.

## 2. Results

### 2.1. Tartary Buckwheat FtMYB3 Is an SG4 R2R3 MYB Protein

**Figure 1:**
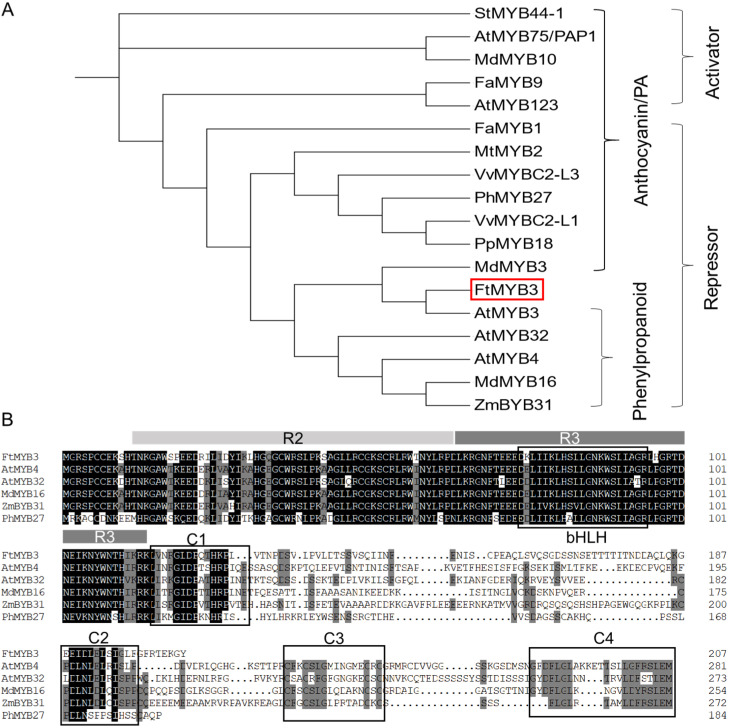
Molecular identification of FtMYB3. (**A**) Phylogenetic tree derived from amino acid sequences of R2R3-MYB TFs in Tartary buckwheat and other species. GenBank accession numbers are as follows: StMYB44-1 (QCH00894.1), AtMYB75 (Q9FE25.1), MdMYB10 (ACQ45201.1), FaMYB9 (JQ989281.1), AtMYB123 (Q9FJA2.1), FaMYB1 (AF401220), MtMYB2 (XP_003616388.1), VvMYBC2-L3 (KM046932), PhMYB27 (AHX24372.1), VvMYBC2-L1 (ABW34393), PpMYB8 (ACA33844.1), MdMYB3 (AEX08668.1), FtMYB3 (AEC32978.1), AtMYB3 (BAA21618.1), AtMYB32 (EFH43356.1), AtMYB4 (BAA21619), MdMYB16 (ADL36756.1), ZmMYB31 (NP_001105949). FtMYB3 is highlighted with a red box. (**B**) Multiple sequence alignments of MYB3. Conserved residues are highlighted in black and partial conservation is shown in grey. The R2 and R3 domains are indicated in greyish white and grey boxes, respectively. Conserved motifs in the C terminus are boxed and numbered as C1~C4. Identical and similar amino acid residues are marked in black and grey, respectively.

Based on the genomic database and our transcriptomic database (data not shown), *FtMYB3* (GenBank ID: JF313349) was identified in Tartary buckwheat. The full-length cDNA of *FtMYB3* is 624 bp in size and encodes a putative protein of 207 amino acids with a predicted molecular weight of about 23.4 kDa and a calculated isoelectric point (PI) of 8.19. Phylogenetic analysis indicated that FtMYB3 was phylogenetically related to AtMYB3 and MdMYB3 (Figure 1A), belonging to the subgroup 4 (SG4) R2R3 family of plant MYB TFs. These factors negatively regulate the *A. thaliana* phenylpropanoid biosynthesis and activate the apple anthocyanin accumulation, respectively [35,36]. Amino acid sequence alignments between FtMYB3 and other MYBs indicated that FtMYB3 consists of R2 and R3 DNA-binding domains at the N terminus and a bHLH motif ([D/E]L × 2[R/K] × 3L × 6L × 3R) within R3 [37] (Figure 1B). Moreover, FtMYB3 had a relatively short C-terminal region with two conserved motifs C1 and C2. These results suggest FtMYB3 is an SG4 R2R3 MYB protein that may function as a transcriptional regulator in the flavonoid pathway.

### 2.2. Expression Pattern of FtMYB3 and Anthocyanin Content in Tartary Buckwheat

**Figure 2:**
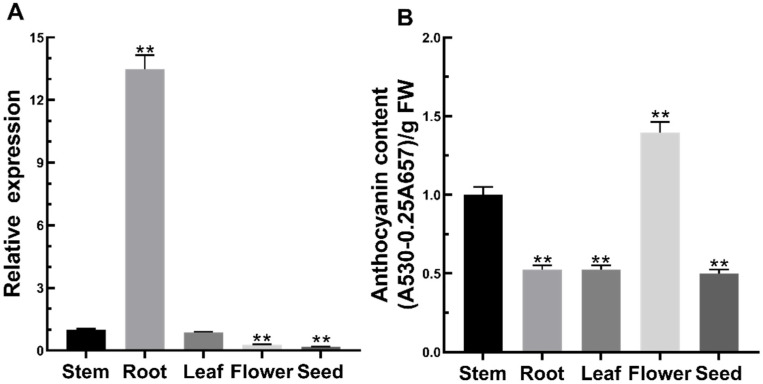
The transcription level of *FtMYB3* and anthocyanin content in different organs. Tartary buckwheat roots, stems, leaves, flowers, and seeds were collected at 40 days after sowing for the analysis of gene expression pattern and the determination of anthocyanin content. (**A**) FtMYB3 expression level in different organs; (**B**) Anthocyanin content in various organs. The accumulation of anthocyanin content and gene expression level in stems are defined as “1”. ** means extremely significant difference at *p* < 0.01 level; Error bars reflect ± SDs.

To preliminarily understand the role of FtMYB3 in Tartary buckwheat, the expression pattern of *FtMYB3* was examined by qRT-PCR in the roots, stems, leaves, flowers, and seeds of Xiqiao No. 2 at the flowering stage. As shown in Figure 2A, the expression of FtMYB3 was most highly expressed in roots, followed by stems and leaves. Differently, the lowest expression was exhibited in flowers and seeds, almost tending to 0. Additionally, the total anthocyanin content of these tissues was tested. The anthocyanin content was determined in all tested tissues with a relatively high accumulation in flowers and stems (Figure 2B). Given that the tissue expression pattern of FtMYB3 is negatively correlated with anthocyanin accumulation (R2 = −0.3751), we speculated that FtMYB3 possibly inhibits anthocyanin synthesis in Tartary buckwheat.

### 2.3. Induction of FtMYB3 by Multiple Environmental Factors and Plant Hormones

The transcription level of MYBs was affected by multiple factors, including environmental factors such as temperature, drought and salinity, as well as plant hormones, MeJA and SA [9,38]. To further examine the response of FtMYB3 to the external environment signals, we cloned its promoter starting from the site of 1986 bp at the upstream of ATG start codon (Supplementary Materials, Text S1). Analysis of the cis-regulatory elements within this promoter showed that these elements were classified into two groups based on their responsive functions: environmental responsive elements including light (e.g., G-box, CACGAC), low temperature (e.g., LTR, CCGAAA), drought (e.g., MBS, CAACTG) and hormone-responsive elements including GA (GARE, TCTGTTG), an auxin (e.g., TGA-element, AACGAC), an SA (e.g., TCA-element, CCATCTTTTT) and MeJA (e.g., CGTCA-motif, CGTCA) (Table S1). Furthermore, several other types of cis-acting elements also exhibited many TATA box, CAAT box, and MYB binding sites (Table S1). These results suggest that distinct environmental factors and plant hormones can regulate the expression of *FtMYB3*.

**Figure 3:**
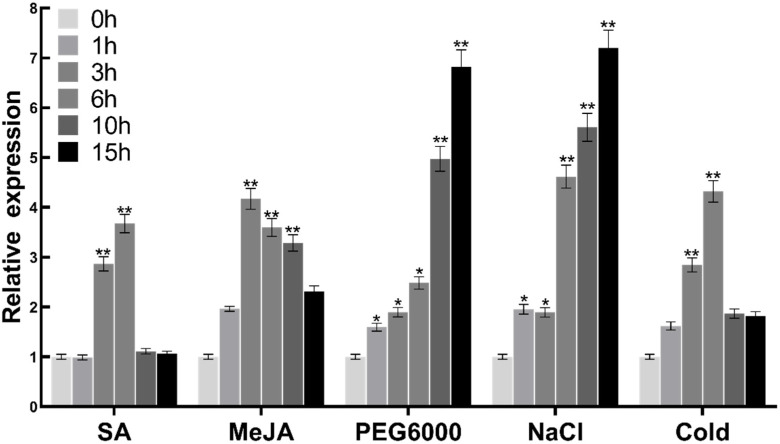
Expression of *FtMYB3* in Tartary buckwheat under different treatments (1 mmol/L SA, 2 mmol/L MeJA, 30% PEG6000, 150 mmol/L NaCl). The samples were collected after respective treatments for 0, 1, 3, 6, 10, and 15 h. The relative expression of FtMYB3 was analyzed by qPCR, and the expression level of *FtMYB3* at 0 h was defined as 1. The expression level was calculated from three replicates. Error bars denote ± SDs. ** means extremely significant difference at *p* < 0.01 level; * means significant difference at *p* < 0.05 level.

qRT-PCR was used to analyze the gene expression pattern of *FtMYB3* in Tartary buckwheat seedlings under different treatments. Under PEG6000 and NaCl treatments, *FtMYB3* expression exhibited a gradually increasing pattern and reached the highest values of 6.82 and 7.19 at 15 h (Figure 3). *FtMYB3* was also strongly induced by SA and MeJA (*p* < 0.01) (Figure 3). Furthermore, low temperature can significantly up-regulate FtMYB3 at 3 h and 6 h. These results show that FtMYB3 may be involved in the stress responses to SA, MeJA, salt, cold and drought.

### 2.4. FtMYB3 Inhibits Anthocyanin/PA Accumulation

**Figure 4:**
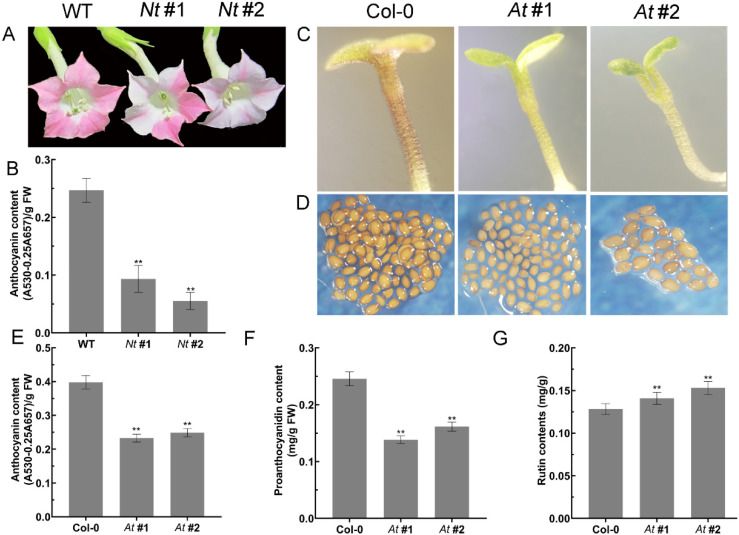
Phenotype of transgenic tobacco (termed *Nt* #1, *Nt* #2) and *Arabidopsis* (named *At* #1, *At* #2) overexpressing *FtMYB3*. (**A**,**B**) Floral phenotypes and total anthocyanin of WT and two transgenic *Nt* #1 and *Nt* #2 at the flowering stage; (**C**) Pigmentation of 6-day-old seedlings in *Arabidopsis* WT (termed Col-0) and transgenic lines *At* #1 and *At #2* after 24 h treatment under 4 °C; (**D**) Colour of seed coats in the same *Arabidopsis* as above without treatments; (**E**–**G**) Anthocyanin, PA and rutin content in the same *Arabidopsis* at the flowering phase. Error bars represent ± SDs. ** means extremely significant difference at *p* < 0.01 level.

To explore whether FtMYB3 regulates anthocyanin synthesis, transgenic tobaccos (termed *Nt* #1, *Nt* #2) and *A.* *arabidopsis* (named *At* #1, *At* #2) overexpressing *FtMYB3* were obtained. Results showed that the petals of lines *Nt* #1 and *Nt* #2 exhibited lighter pigmentation compared with that in wild-type (WT), indicating that the anthocyanin synthesis was inhibited (Figure 4A). Furthermore, the anthocyanin content of tobacco flowers overexpressing *FtMYB3* was significantly decreased by 38.75% and 21.25% compared to that in WT (Figure 4B).

Similarly, the pigment deposition in seedlings of At #1 and At #2 was less than that in *Arabidopsis* WT (named Col-0) (Figure 4C). Moreover, the color of the seed coats was obviously lighter in At #1 and At #2 than in Col-0, indicating weaker pigmentation in plants overexpressing FtMYB3 (Figure 4D). The above results suggest that FtMYB3 may also affect the synthesis of other secondary metabolites. Therefore, the contents of anthocyanin, PA and rutin was determined in transgenic *Arabidopsis*. Figure 4E–G showed that the contents of anthocyanin and PA were significantly decreased in all transgenic plants compared to Col-0. However, the rutin content of lines At #1 and At #2 was significantly higher than Col-0, which was 109.8% and 119.3% of WT, respectively (Figure 4E–G). Collectively, these results suggest that ectopic expression of FtMYB3 inhibits anthocyanin/PA accumulation in plants.

### 2.5. Downregulation of FtMYB3 in the Expression Levels of DFR/BAN/ANS/TT13

**Figure 5:**
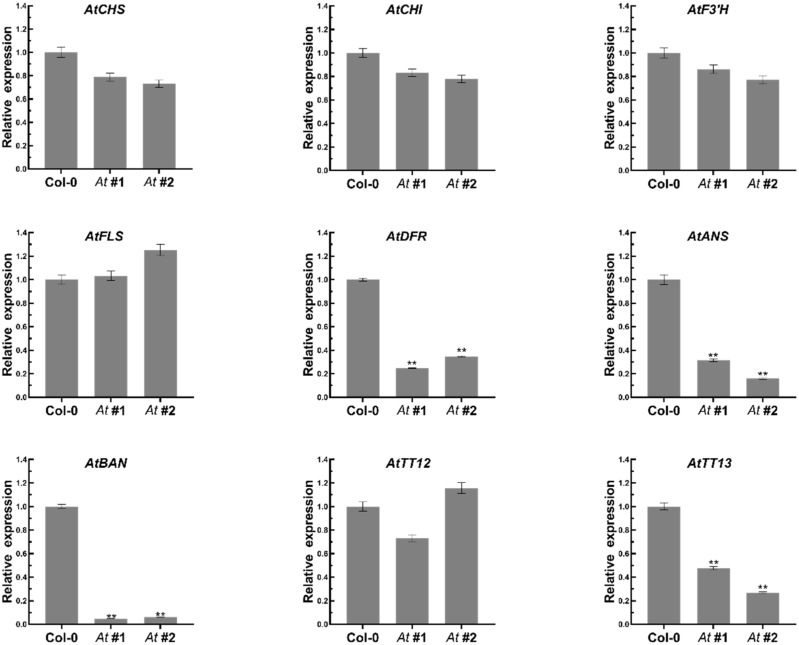
The expression level of enzyme genes in the flavonoid metabolic pathway in *Arabidopsis* overexpressing FtMYB3 (At #1 and At #2) compared with those in Col-0. The mRNA abundances of 9 genes were monitored by qRT-PCR in the 4-week-old seedings of Col-0, At #1, and At #2, respectively. The 2^−ΔΔCT^ method was used to evaluate the relative expression, and AtActin2 was used as a reference gene. Error bars represent ± SDs. ** means extremely significant difference at *p* < 0.01 level.

To elucidate the possible mechanisms of FtMYB3-mediated anthocyanin/PA biosynthesis, a qRT-PCR assay was performed to examine the effects of FtMYB3 on the transcript levels of genes functioning in the flavonoid biosynthetic pathway. Because of a long storage time, seeds of the previous transgenic tobacco failed to germinate. Thus, transgenic *A.* *arabidopsis*, *At* #1 and *At* #2 were used for subsequent experiments. qPCR results indicated that the expression levels of EBG genes (*AtCHS*, *AtCHI*, and *AtF3′H*) were not significantly different in lines *At* #1 and *At* #2 compared to Col-0 (Figure 5). However, the transcript levels of LBG including *DFR*, *ANS* and *BAN* were significantly down-regulated in lines *At* #1 and *At* #2. Moreover, the transcript level of *AtTT13*, which is involved in PA transport [39], was also down-regulated. Interestingly, *AtFLS* showed an elevated expression level in line *At* #2. These results indicated that FtMYB3 regulates multiple structural genes involved in flavonol biosynthesis.

### 2.6. FtMYB3 Interacts with GL3/EGL3/TT8/TTG1

**Figure 6:**
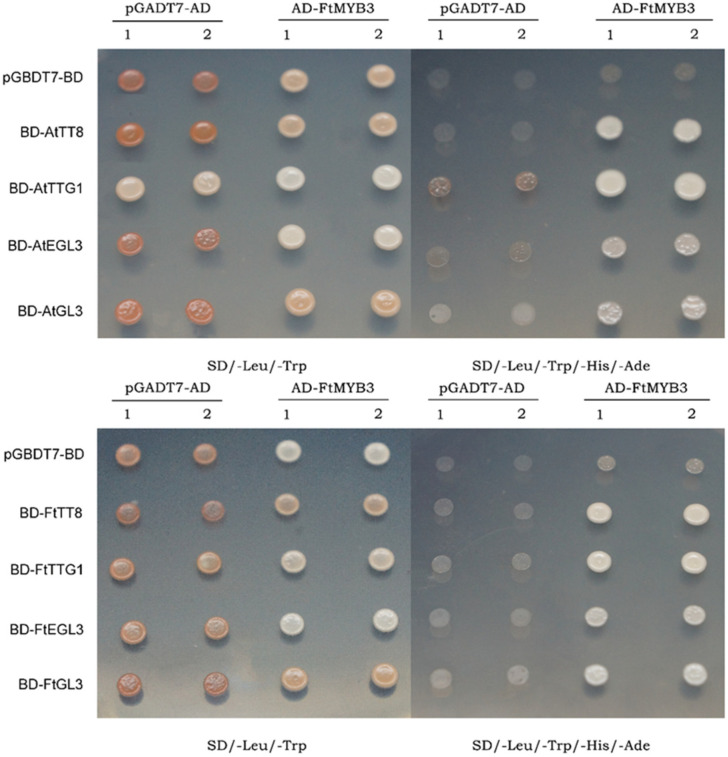
Interaction proteins of FtMYB3. FtMYB3 was fused to the pGADT7 vector, and FtTT8, FtTTG1, FtEGL3, or FtGL3 was fused to the pGBKT7 vector. SD/-Trp-Leu medium was used to screen the transformation for plasmids, and SD/-Leu-Leu-His-Ade medium was used to screen the transcriptional activation of *HIS3* gene. Colony growth was monitored after 7 days. Yeast cells transformed with the empty plasmids pGADT7 and pGBKT7 were used as controls, respectively. Two independent technical replicates of each strain were analyzed.

Previous studies have shown that MYB-bHLH-WD repeat (MBW) complexes regulate the transcription of anthocyanin genes in plants [27]. Y2H was performed to screen interacting proteins of FtMYB3 involved in the formation of MBW complex. As shown in Figure 6, FtMYB3 can interact with GL3/EGL3/TT8/TTG1 in Tartary buckwheat and *Arabidopsis*, which suggests that FtMYB3 might regulate the transcriptional expression of key enzyme genes in the pathway of anthocyanin synthesis.

### 2.7. FtMYB3 Inhibits the Transcriptional Activation Activity of the DFR Promoter

**Figure 7:**
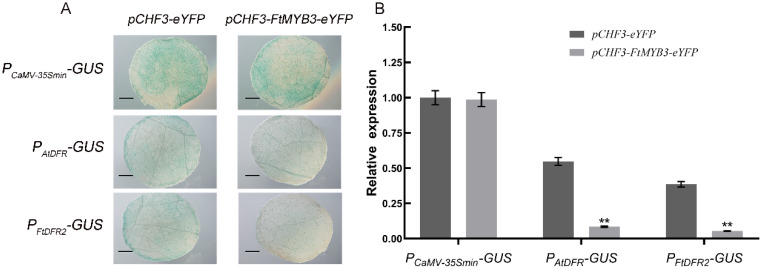
Inhibition of FtMYB3 on the *DFR* promoter in instantaneous transfected tobacco producing FtMYB3-eYFP together with the *GUS* gene under the control of the *P_CaMV-35Smin_-GUS*, *P_AtDFR_-GUS* and *P_FtDFR2_-GUS*, respectively. (**A**) Staining of GUS protein. Tobacco leaves that were about 4 weeks old were used for transient transformation analysis, and GUS protein was stained 48 h after injection; (**B**) Relative expression of *GUS* gene. Error bars indicate ± SDs. ** means significant difference at *p* < 0.01 level.

*DFR* is one of the essential enzyme genes for the synthesis of anthocyanin and flavonol branches. A transient expression assay was designed to test the impact of FtMYB3 on the transcriptional activation activity of P*_AtDFR_* and P*_FtDFR2_*. As shown in Figure 7, GUS staining analysis showed that expression of the reporter gene declined significantly in the samples co-transformed with pCHF3-FtMYB3-eYFP. qRT-PCR detected the transcription level of the target gene *GUS* and showed that it had little or no transcriptional expression in the experimental group, suggesting that *FtMYB3* indeed represses the transcriptional activation activity of *P_AtDFR_* and *P_FtDFR2_*.

## 3. Discussion

To date, most of the R2R3 MYB TFs have been well demonstrated to play a regulatory role in the flavonoid pathway in distinct plants. However, only two MYB repressors involved in the anthocyanin and PA biosynthesis were identified in Tartary buckwheat. In this study, we reported a novel R2R3-MYB TF FtMYB3 that negatively regulates anthocyanin and PA biosynthesis in a direct way.

### 3.1. FtMYB3 Is a Special SG4 MYB Repressor That Reduces Anthocyanin and PA Accumulation

In *Arabidopsis*, more than 100 R2R3-MYB TFs were divided into 25 sub-groups (SGs) [40]. Numerous R2R3-MYB TFs have been shown to be involved in the control of plant primary and secondary metabolism. Notably, SG4 and SG4-like are major TFs coordinating anthocyanin and PA biosynthesis. SG4 contains the conserved C2 motif that represses phenylpropanoid, sinapate ester, and flavonoid biosynthesis, while SG4-like contains the conserved C5 motif that participates in anthocyanin and PA biosynthesis. For instance, it has been shown that overexpression of *AtMYB3*, a SG 4 MYB protein represses phenylpropanoids biosynthesis [35]. The phylogenetic analysis indicated that FtMYB3 is very closely related to AtMYB3. In this study, we found that both anthocyanin and PA contents were significantly lower in transgenic *Arabidopsis* than Col-0, which suggests that the *FtMYB3* gene is also involved in the negative regulation of anthocyanin and PA. In addition, *FtMYB3* is also a homologue to the apple *MdMYB3*. Differently, ectopic expression of MdMYB3 in tobacco promotes anthocyanin and flavonol accumulation, even though these three genes contain the conserved C2 motifs at the C-terminus [36]. However, the functional differences among FtMYB3, MdMYB3, and AtMYB3 remain unclear.

In Tartary buckwheat, two SG4-like R2R3 TFs FtMYB8 and FtMYB18 play a role in the synthesis of anthocyanins and Pas [31,32]. Their C-terminus contains a C5 motif, which is identified as the key to inhibiting the synthesis of Tartary buckwheat anthocyanins and Pas [31]. Similarly, other SG4-like also have an inhibitory effect on anthocyanin and PA bio-synthesis. For example, MrMYB6 might negatively regulate anthocyanin and PA ac-cumulation in Chinese bayberry [41]. A similar finding was observed when PtrMYB57 was overexpressed in poplar [42]. Interestingly, we have observed a significant increase in rutin content in *Arabidopsis* upon overexpression of FtMYB3, indicating that other substrates can be involved in rutin synthesis. Many studies have reported a competitive relationship between the synthesis of anthocyanins and flavonols [43,44]. Overall, these results reveal that *FtMYB3* may be a repressor for anthocyanin and PA biosynthesis.

### 3.2. FtMYB3 Specifically Represses the Expression of Genes in the Late Pathway of Flavonoid Synthesis

Previous studies have shown that the accumulation of anthocyanins and PAs are controlled by transcriptional regulation of genes encoding the early and late biosynthetic enzymes. In *Ginkgo biloba*, GbMYBF2 specifically inhibited the flux of flavonoid by repressing the transcription of *CHS*, *F3H*, *FLS*, and *ANS*, resulting in a decrease in anthocyanin contents [45]. Similarly, a C2 suppressor NtMYB2 from Chinese Narcissus inhibits the expression of key enzyme genes such as *CHS*, *F3H*, *DFR*, *FLS*, and *ANS* in transgenic tobacco, subsequently inhibiting the synthesis of anthocyanin and flavonol [46]. Moreover, FtMYB18 from Tartary buckwheat regulates anthocyanin synthesis by inhibition of EBGs (*FtCHS*) and LBGs (*FtDFR*) [31]. However, the inhibition of FtMYB8 in *TTI12* to regulate anthocyanin/PA synthesis was mediated by anthocyanin/PA transport rather than anthocyanin/PA synthesis-related genes [32]. In this study, *FtMYB3* significantly repressed the transcription level of *LBGs* (*DFR*, *ANS* and *BAN*) upon its overexpression in *Arabidopsis* (*p* < 0.01). Remarkably, we also noted the reduced transcript abundance of an anthocyanin transporter gene *TT13*, which functions as a proton pump in the tonoplast of seed coat endothelium cells [39]. Given that *FtMYB3* may have unique downstream target genes, we speculate that FtMYB3 may have a distinct regulation mechanism compared with previous anthocyanin and PA inhibitors.

### 3.3. Possible Mechanism of FtMYB3 Inhibiting the Biosynthesis of Anthocyanins and PAs

R2R3-MYB repressors mainly include two types, FaMYB1-like and AtMYB4-like [47]. FaMYB1-like repressors incorporate into or bind MBW complexes to repress genes. AtMYB4-like repressors directly bind on the promoter of target genes to actively repress transcription through its C terminal motifs such as C2, C3 or C4. For example, *MdMYB16* directly inhibited the expression of *MdUFGT* and *MdANS* via its C2 motif in apple [16]. The *Arabidopsis* R3 type MYB transcription factor AtCPC also forms a complex to occupy the PAP1/2 binding sites of proteins such as TTG1, GL3, and EGL3, which inhibits the expression of late enzyme genes in the anthocyanin synthesis pathway and reduces anthocyanin synthesis and accumulation [48]. In this study, FtMYB3 was confirmed to interact with FtTT8, FtGL3, FtEGL3 and FtTTG1 in vitro, thereby inhibiting the transcriptional expression of vital enzyme genes in the anthocyanin pathway. We also found that FtMYB3 represses the transcriptional activation activity of *P_AtDFR_* and *P_FtDFR2_*, indicating that FtMYB3 may also directly target these genes. In addition, our previous studies have shown that FtMYB3 can interact with the jasmonate-ZIM domain protein FtJAZ2 to affect anthocyanin biosynthesis [33]. Notably, we found that *FtMYB3* was strongly induced by MeJA. These results suggest that FtMYB3 regulates anthocyanin and PA biosynthesis through multiple pathways.

## 4. Materials and Methods

### 4.1. Plant Materials and Growth Conditions

The Tartary buckwheat “Xiqiao No. 2” used in this study is from Professor Anhu Wang of Xichang University. The *A. thaliana* ecotype Columbia-0 (Col-0) was provided by Professor Yi Cai of Sichuan Agricultural University and the *Nicotiana tabacum* (NC89) was obtained from Professor Jinwen Zhang of Gansu Agricultural University. Different tissues (roots, stems, leaves, flowers, and seeds) from “Xiqiao No. 2” were collected at the flowering stage. Three biological replicates were prepared for each sample and immediately frozen in liquid nitrogen at −80 °C for storage.

### 4.2. Total RNA Extraction and Gene Expression Analysis

Total RNA from various plants was extracted using the EASYspin Plant RNA Kit (Aidlab, Beijing, China). Elimination of genomic DNA and the synthesis of first-strand cDNA were performed using the PrimeScript™ RT reagent Kit with gDNA Eraser (Takara, Dalian, China). qRT-PCR was conducted using SYBR^®^ Premix Ex TaqTM II (Takara Bio Inc., Dalian, China), and the PCR program was as follows: one cycle of 3 min at 95 °C, followed by 40 cycles of 10 s at 95 °C and 30 s at 60 °C. Three genes, *FtH3*, *AtActin2* and *Ntβ-actin* were used as an internal control for Tartary buckwheat, *Arabidopsis* and tobacco, respectively. The relative expression level of these genes in this study was calculated using the Change 2^(−ΔΔCt)^ method. All analyses were conducted with three biological replicates for each sample. Primer sequences used for qRT-PCR are listed in Table S2.

### 4.3. Sequence Analysis of FtMYB3 and Its Promoter

The full-length open reading frame (ORF) of *FtMYB3* was obtained by PCR using gene-specific primers in terms of the published sequence (GenBank ID: JF313349). The molecular weight, isoelectric point (pI), and theoretical coding sequence of FtMYB3 were predicted using an online ExPASy server: https://web.expasy.org/protparam/ (accessed on 7 January 2022). The protein sequences of R2R3-MYB transcriptional factors from other species were collected from NCBI: https://blast.ncbi.nlm.nih.gov/Blast.cgi (accessed on 20 August 2020). Subsequently, these sequences were aligned with FtMYB3 by the DNAMAN6.0 software with default parameters. The entire protein sequence of R2R3-MYB TFs related to FtMYB3 were used to create a phylogenetic tree using MEGA5 software with default parameters. The bootstrap was 1000.

### 4.4. Construction of Expression Vectors and Stable Transformation of Arabidopsis and Tobacco

To obtain the vector pCHF3-FtMYB3-eYFP, PCR was performed with primers FtMYB3F and FtMYB3R using cDNA as a template. The cDNA was synthesized based on RNA extracted from young leaves of “Xiqiao No. 2”. PCR amplification was performed using the KOD-plus-neo high fidelity enzyme kit (Toyobo, Osaka, Japan). The PCR fragment without the stop codon encoding the whole coding sequences of *FtMYB3* was inserted between *Kpn* I and *Sal* I sites in plasmid pCHF3-YFP that was provided by Professor Yi Cai of Sichuan Agricultural University. Subsequently, the pCHF3-FtMYB3-eYFP vector was introduced into Col-0 through *Agrobacterium tumefaciens* GV3101 by the floral dip method [49]. Meanwhile, pCHF3-FtMYB3-YFP was transformed into NC89 by using the Agrobacterium strain LBA4404 according to the method described by Huang [32]. The first generation (T1) seeds of transgenic plants were collected and screened in 1/2 MS medium with 50 mg L-1 kanamycin. Next, the kanamycin-resistant plants were transferred into soil pots and grown in a growth chamber with a 16 h photoperiod cycle at 25 °C. Similarly, the second generation (T2) seeds were collected. Finally, the T3 homozygous positive lines were collected and used for subsequent experiments. Primer sequences used for constructing the expression vector are listed in Table S2.

### 4.5. Determination of Total Anthocyanin, PA and Rutin Content

Total anthocyanins were extracted and quantified in fresh leaves of transgenic *Arabidopsis* using the previously described method in [50] as follows: (1) the samples were ground in liquid nitrogen to produce powder; (2) 200 mg powder were used to extract the anthocyanins in 1 mL of an acidic methanol solution; (3) the above solution was sedimented by centrifugation (12,000× *g*, 4 °C, 1 min) and 400 μL of the supernatant was added to 600 μL of acidic methanol and then filtered over a 0.2 μm Teflon filter before analysis; and (4) the anthocyanins were then measured at 530 and 657 nm in a spectrometer (UNICO WFJ2000, China). The extraction and measurement of Pas were carried out as previously described in [51]. High-performance liquid chromatography (HPLC) was performed to determine the rutin content according to the description from Yao [52].

### 4.6. Yeast Two-Hybrid Assay (Y2H)

For Y2H assay, the full-length CDS of *FtMYB3* was inserted into the pGADT7 vector as a bait vector. Meanwhile, the full-length CDS of *AtGL3*, *AtEGL3*, *AtTTG1*, *FtTT8*, *FtGL3*, *FtEGL3* and *FtTTG1* was inserted into the pGBKT7 vector as a prey vector. Subsequently, the prey and the bait constructs were transformed into yeast strain AH109 (Clontech) [53]. The yeast cells were grown on SD/-Trp-Leu medium after mating. Positive colonies were confirmed by PCR and further grown on SD/-Trp-Leu-His-Ade defective medium. Pictures were captured on day 3 after incubation. pGADT7 and pGBKT7 were provided by Professor Yi Cai of Sichuan Agricultural University. All primers used for PCR are listed in Table S2.

### 4.7. Transient Expression Assay

The promoter of *FtDFR2* and *AtDFR* was inserted into the plant expression vector pCAMBIA1304 by replacing the 35S promoter before the *GUS* gene, respectively. The recombinant vector pCAMBIA1304-*P_FtDFR2_*-*GUS* (termed *P_FtDFR2_-GUS*) and pCAMBIA1304-*P_AtDFR_*-*GUS* (termed *P_AtDFR_-GUS*) were transformed into the GV3101 and then injected into the young leaves of tobacco expressing pCHF3-eYFP or pCHF3-FtMYB3-eYFP vectors, respectively. Lastly, the expression of *GUS* gene was detected by qRT-PCR and visualized by histochemical staining after 48 h incubation.

### 4.8. Statistical Analysis

Statistical analyses were conducted by SPSS 22.0 software. All data were obtained from three biological replicates. * and ** indicate *p*-values < 0.05 and <0.01, respectively.

## 5. Conclusions

**Figure 8:**
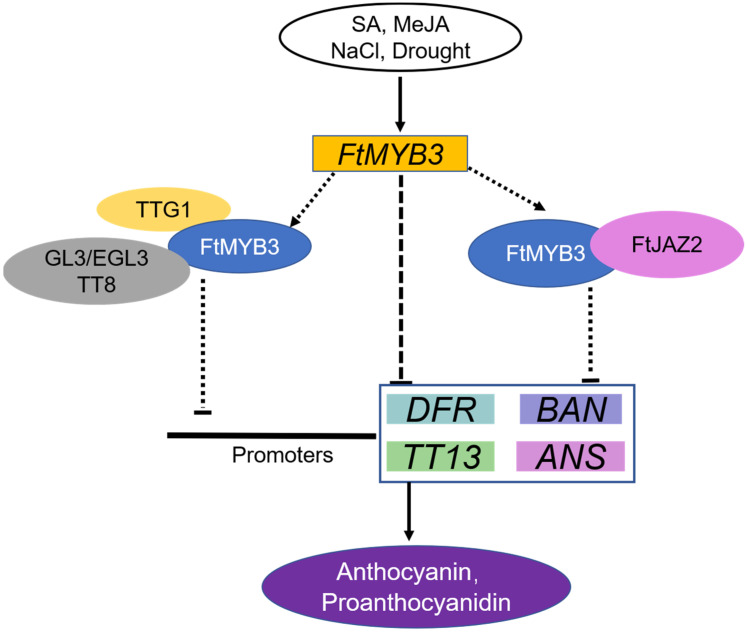
A proposed model depicting FtMYB3 regulation of anthocyanin and PA synthesis under different stresses. FtMYB3 is induced by SA, MeJA, NaCl and drought stress. FtMYB3 directly interacts with TT8, GL3, EGL3, and TTG1 and suppresses their transcriptional activator of *DFR*, *ANS*, *BAN* and *TT13*. Additionally, FtMYB3 may inhibit the synthesis of anthocyanins/PAs by interacting with FtJAZ2 protein.

In this study, our data indicate that FtMYB3 is an R2R3 inhibitor that negatively regulates the anthocyanin and PA accumulation by the strong downregulation of *DFR*, *ANS*, *BAN* and *TT13*. Therefore, *FtMYB3* may be a potential target gene for improving the quality of Tartary buckwheat. Furthermore, we found that FtMYB3 can directly interact with TT8, GL3, EGL3, and TTG1 in vitro. Based on these results, we propose a putative model in which FtMYB3 accumulation leads to decreased expression of *DFR* and *ANS* and thus reduces anthocyanin and PA content (Figure 8). Taken together, this study promotes further understanding of the molecular mechanisms of regulation for anthocyanin and PA synthesis in Tartary buckwheat.

## Data Availability

Not applicable.

## Supplementary Materials

The following supporting information can be downloaded at: https://www.mdpi.com/article/10.3390/ijms23052775/s1.
